# Neural correlates of acute post-traumatic dissociation: a functional neuroimaging script-driven imagery study

**DOI:** 10.1192/bjo.2022.65

**Published:** 2022-06-10

**Authors:** Yoki L. Mertens, Antje Manthey, Anika Sierk, Henrik Walter, Judith K. Daniels

**Affiliations:** Department of Clinical Psychology and Experimental Psychopathology, University of Groningen, The Netherlands; Charité University Clinic Berlin, Freie Universität Berlin, Humboldt-Universität zu Berlin and Berlin Institute of Health, Germany; Charité University Clinic Berlin, Freie Universität Berlin, Humboldt-Universität zu Berlin and Berlin Institute of Health, Germany; Charité University Clinic Berlin, Freie Universität Berlin, Humboldt-Universität zu Berlin and Berlin Institute of Health, Germany; Department of Clinical Psychology and Experimental Psychopathology, University of Groningen, The Netherlands

**Keywords:** Post-traumatic stress disorder, imaging, trauma confrontation, dissociation, biomarkers

## Abstract

**Background:**

Current neurobiological models of post-traumatic stress disorder (PTSD) assume excessive medial frontal activation and hypoactivation of cortico-limbic regions as neural markers of post-traumatic dissociation. Script-driven imagery is an established experimental paradigm that is used to study acute dissociative reactions during trauma exposure. However, there is a scarcity of experimental research investigating neural markers of dissociation; findings from existing script-driven neuroimaging studies are inconsistent and based on small sample sizes.

**Aims:**

The current aim was to identify the neural correlates of acute post-traumatic dissociation by employing the script-driven imagery paradigm in combination with functional magnetic resonance imaging.

**Method:**

Functional neuroimaging data was acquired in 51 female patients with PTSD with a history of interpersonal childhood trauma. Blood-oxygen-level-dependent response during the traumatic (versus neutral) autobiographical memory recall was analysed, and the derived activation clusters were correlated with dissociation measures.

**Results:**

During trauma recall, enhanced activation in the cerebellum, occipital gyri, supramarginal gyrus and amygdala was identified. None of the derived clusters correlated significantly with dissociative symptoms, although patients reported increased levels of acute dissociation following the paradigm.

**Conclusions:**

The present study is one of the largest functional magnetic resonance imaging investigations of dissociative neural biomarkers in patients with PTSD undergoing experimentally induced trauma confrontation to elicit symptom-specific brain reactivity. In light of the current reproducibility crisis prominent in neuroimaging research owing to costly and time-consuming data acquisition, the current (null) findings highlight the difficulty of extracting reliable neurobiological biomarkers for complex subjective experiences such as dissociation.

Post-traumatic dissociation – experiencing depersonalisation or derealisation sensations, often in response to stressors or trauma reminders – occurs frequently in people suffering from post-traumatic stress disorder (PTSD).^1^ Despite the increased attention paid to post-traumatic dissociation following the inclusion of a dissociative PTSD subtype in the DSM-5,^2^ neuroimaging research on the topic remains scarce. Recently published systematic reviews indicate that only a few dissociation-specific neural alterations could be identified in patients with PTSD, with most of them awaiting independent replication.^3,4^ The majority of the existing literature reported brain activation during the resting state by comparing subgroups of patients based on their long-term patterns of responding without inducing a dissociative state during the actual data acquisition. Across studies, tentative support exists that patients with dissociative PTSD, compared with the classic PTSD group, depict increased connectivity between (pre-)frontal regions and subcortical regions, such as the amygdala, the periaqueductal gray^5^ and the cerebellum.^6^ The temporoparietal junction, a critical hub for multisensory integration, has shown increased connectivity to the superior colliculus^7^ as well as the periaqueductal gray.^8^ Altered brain circuity was further reported between the right supramarginal gyrus and the thalamus,^9^ of which the latter region depicted lower fractional anisotropy in a network with the left amygdala and hippocampus, correlating negatively with self-reported depersonalisation experiences.^10^ Such brain activation patterns during the resting state might detect differential connectivity networks, but cannot establish direct causal relationships between the observed alterations and the occurrence of dissociative symptoms. To achieve better causal attribution, symptom induction during data acquisition is needed.

## Script-elicited neural correlates of dissociation

The most well-established symptom provocation paradigm in PTSD neuroimaging research is script-driven imagery (SDI).^11,12^ During SDI, participants are asked to repeatedly listen to and actively recall autobiographical memory audio scripts. According to a recent meta-analysis on the neural underpinnings of trauma-related autobiographical memory recall,^13^ different mental processes may underly the retrieval of memory versus active engagement during imagery. These processes might correspond to different parts of the SDI paradigm: during the first 30 s, retrieval is prompted by passively listening to the script, while the patient is asked to actively imagine the scene described in the script for the following 30 s, and are assumed to correspond with differential brain activation patterns in PTSD.

To date, only a few studies have investigated neural activation patterns associated with the experience of post-traumatic dissociation by using the SDI paradigm. Early studies employing the SDI were severely underpowered (*n* = 2^14^ and *n* = 7^15^ participants with PTSD), but introduced the idea of an absent amygdala activation on the backdrop of excessive prefrontal regulation as a correlate of dissociation. Lanius et al later suggested that such an overmodulation of the limbic system might be the causal mechanism differentiating the dissociative subtype of PTSD.^16^ Hopper et al^17^ employed SDI in 27 (mostly female) patients with chronic PTSD following (predominantly) motor vehicle accidents. The participants reported the severity of acute dissociation in response to the trauma confrontation directly following the functional neuroimaging paradigm. Dissociation correlated positively with activity in the left medial prefrontal and right superior temporal cortices, and negatively with the left superior temporal cortex and the right anterior insula. The anterior insula subserves interoception of bodily states and its underactivation in patients with dissociation was interpreted as ‘biological substrate of extreme emotional underengagement’.^17^ However, these correlates could not be replicated by the following two studies from the same laboratory also employing the SDI paradigm. One found positive correlations between acute dissociation and activation of the left middle frontal gyrus and right superior frontal gyrus (*N* = 21^18^) in a sample of participants with acute trauma. No significant neural correlates of acute dissociation were identified in a study probing pain processing with the SDI in PTSD (*N* = 17).^19^ Instead, a negative correlation between trait dissociation and activity in the amygdala, putamen, anterior cingulate cortex and left superior frontal gyrus was established.^20,21^ In patients suffering from borderline personality disorder, dissociative processing during the SDI was associated with greater activation of the left superior frontal gyrus and lower activation of the right middle and inferior temporal gyri,^22^ whereas Krause-Utz et al reported diminished bilateral amygdala activation as a correlate of acute dissociation after participants underwent a SDI task.^23^

In sum, there is some, albeit inconsistent, fMRI evidence suggesting that post-traumatic dissociation may be related to a fronto-limbic imbalance concurrent with increased feelings of depersonalisation, derealisation and emotional numbing reported by patients with PTSD. However, the heterogeneity of the results so far precludes the identification of a biomarker of post-traumatic dissociation. This heterogeneity may be because of various factors, including differences in sample composition (e.g. trauma event type, time since trauma exposure), usage of different measures capturing alternative aspects of dissociative experiencing (e.g. identity confusion) and small sample sizes. The field of neuroimaging has seen a lot of criticism regarding the low statistical power of most studies, because of their small sample sizes,^24^ and simulation studies have shown that samples with less than 30 participants – such as the ones cited above – show particularly large variation on subsequent replications.^25^ Because of the high costs of neuroimaging, concerted efforts are needed to (a) provide single studies with adequate sample sizes analysed, using power-enhancing analysis strategies; and (b) pool data across studies as recently done within large consortiums (e.g. Wang et al^26^). However, to date, neither option has been applied to the identification of biomarkers of post-traumatic dissociation.

## Study aims

The current study aims to identify neural activation patterns associated with acute post-traumatic dissociation during symptom provocation. To this end, a large sample (*N* > 50) of patients with PTSD with coherent characteristics (female gender, history of interpersonal childhood trauma) will be analysed. The secondary aim of this study is to evaluate the paradigm most often used in the field, i.e. the SDI paradigm regarding the distinction of underlying memory processes and its usefulness for the identification of biomarkers of dissociation.

## Method

### Participants

A total of *N* = 51 female participants (mean age 40.29 years, s.d. 10.17) diagnosed with PTSD were included in the current study. Participants were recruited by means of public advertisements and mental health treatment centres. Interested individuals were first screened via telephone for magnetic resonance imaging (MRI) compatibility and medication status. They were fully informed about the study procedure and potential risks before signing the informed consent. Written informed consent was obtained from all participants. They received a booklet containing the self-report instruments and were asked to fill these in at home. At least one full day before the scanning session, interested patients were invited to the laboratory to undergo clinical diagnostics. They were included in the study if they met all inclusion criteria: 20–60 years, proficient in German, MRI compatible, no neurological disorder, no history of head injury, no substance dependency in the past 6 months, no intake of benzodiazepine or anticonvulsants, PTSD as primary disorder, PTSD symptoms for at least 3 months and experience of interpersonal childhood trauma before the age of 22 years. To account for the high comorbidity encountered in PTSD, participants with the following comorbid disorders as a secondary diagnosis were eligible: comorbid depressive, anxiety, eating, substance misuse and borderline personality disorders. Patients with other comorbidities (e.g. dissociative disorders) were excluded.

The current data stems from a larger trial investigating medication effects. Participants were scanned twice on two separate days. For the current article, only data from the placebo condition were used, which was counterbalanced across participants. During data collection, a single participant dropped out (owing to an accident) in between sessions, but their data from the first day of scanning (placebo condition) could be included in is the current analysis. All other participants completed their participation; however, data for one participant could not be included for current analysis because of missing log files, resulting in a total sample of *N* = 51.

The authors assert that all procedures contributing to this work comply with the ethical standards of the relevant national and institutional committees on human experimentation and with the Helsinki Declaration of 1975, as revised in 2008. The study was approved by the medical ethics board of the University of Magdeburg [57/14] and the ethical committee of the Berlin Psychological University, and was preregistered (aspredicted.org: 78135).

### Procedure

After study inclusion, the scripts employed during the SDI paradigm were developed. To this end, participants were asked to provide short but detailed narratives (told from a first-person perspective in the present tense) of one neutral and one traumatic autobiographical memory containing specifically associations (e.g. time of day, bodily sensations, sounds, smell, colours, mood, cognition) to facilitate recollection and re-experiencing during script exposure. The trauma scripts were based on the traumatic memory that elicited the strongest intrusion symptoms at the point of testing (without being too overwhelming). In the current sample, the majority of trauma scripts related to experiences of childhood trauma (80.4%), of which 76.5% related to childhood sexual abuse, physical abuse or a combination of both (see Table 1). The neutral scripts were based on recent adult memories regarding everyday situations without a specific positive or negative valence (e.g. doing laundry). The narratives were converted into audio scripts with a neutral voice. In the days following script collection, participants were invited to the laboratory to undergo neuroimaging testing.

**Table 1:** Demographic variables

	% (*n*)	Mean (s.d.)
Age, years		40.29 (10.17)
School education (highest degree)		
High school diploma	82.3 (42)	
Junior high school diploma	17.6 (9)	
Work experience (highest degree)		
University degree	45.1 (23)	
Vocational training/apprenticeship	31.4 (16)	
Trainee	11.8 (6)	
None	11.8 (6)	
Relationship status		
Single	51.0 (26)	
In partnership	21.5 (11)	
Married	19.6 (10)	
Divorced	21.6 (11)	
Widowed	2.0 (1)	
Lifetime traumatic experiences (ETI-TL)		
Childhood sexual abuse^a^	84.3 (43)	
Violent attack^a^	78.4 (40)	
Neglect	68.6 (35)	
Severe illness	60.8 (31)	
Sexual abuse in adulthood^a^	52.9 (27)	
Death of close person	49.0 (25)	
Accidental trauma	43.1 (22)	
Torture	17.6 (9)	
Natural disaster	17.6 (9)	
Imprisonment	11.8 (6)	
War combat	3.9 (2)	
Age at onset of worst traumatic experience	17.61 (12.37)	
Interpersonal childhood trauma (CTQ)		
Childhood sexual abuse (≥ 8)	72.5 (37)	14.10 (7.25)
Childhood physical abuse (≥ 8)	60.8 (31)	11.12 (5.73)
Childhood emotional abuse (≥ 10)	82.4 (42)	19.06 (5.61)
Childhood emotional neglect (≥ 15)	78.4 (40)	18.56 (4.79)
Childhood physical neglect (≥ 8)	76.5 (39)	11.58 (4.70)
Above cut-off on any abuse subscale	91.7 (44)	2.29 (0.97)
Above cut-off on any neglect subscale	91.7 (44)	1.65 (0.64)
Above any cut-off (combined)	95.8 (46)	3.94 (1.39)
Traumatic memory for script^b^		
Childhood trauma	80.4 (41)	
Childhood sexual abuse	54.9 (28)	
Childhood physical abuse	11.8 (6)	
Childhood combined sexual and physical abuse	9.8 (5)	
Other	3.9 (2)	
Adult trauma	19.6 (10)	
Adult sexual abuse	5.9 (3)	
Adult physical abuse	3.9 (2)	
Other	5.9 (3)	
Comorbid diagnosis (SCID-I and -II)		
Anxiety disorder	80.4 (41)	
Affective disorders	41.2 (21)	
Borderline personality disorder	15.7 (8)	
Eating disorders	9.8 (5)	
Obsessive–compulsive disorder	6.0 (3)	
Substance misuse	2.0 (1)	

ETI-TL, Essen Trauma-Inventory – Trauma Checklist (multiple answers possible); CTQ, Childhood Trauma Questionnaire; SCID-I and -II, Structured Clinical Interview for DSM-IV Axis I and Axis II Disorders.

a. Perpetrated by familiar person (e.g. family member), stranger or both.

b. Defined as traumatic memory eliciting strongest intrusion symptoms at assessment.

**Fig. 1.**
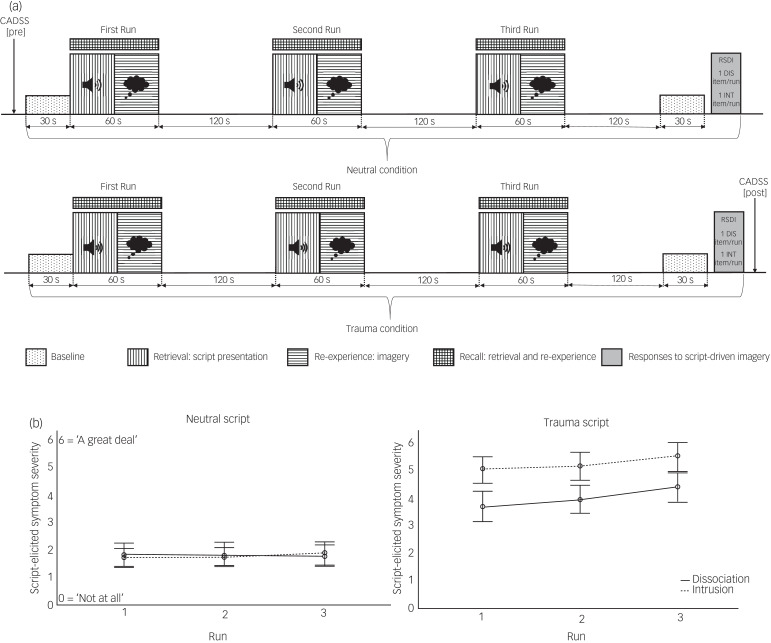
Script-driven Imagery Paradigm. a, Schematic presentation of script-driven imagery paradigm. b, Script-elicited dissociation and intrusion for each run per script (Error bars: 95% Confidence Interval) CADSS, Clinician-Administered Dissociative States Scale; DIS, Dissociation; INT, Intrusion; RSDI, Responses to Script-driven Imagery Scale.

The functional imaging paradigm consisted of two condition blocks (neutral and trauma SDI), each comprising a 30-s baseline period at the beginning as well as the end of the block, and three runs (repetitions of the script presentation). Each run lasted 3 min in total: 1 minute of imagery period (27 s of listening to the audio script and imaging event, and 33.75 s active recall) followed by 2 min of a recovery period, indicated by a bell sound. Congruent with previous research, the neutral condition always preceded the trauma condition to avoid carry-over effects (see Mickleborough et al^19^). At the end of each block, participants completed a self-report scale^27^ to assess self-reported intrusive, avoidant, and dissociative responding elicited during the three recall periods across each block. Additionally, one re-experiencing (‘During run X, did you re-experience part of the trauma involuntarily (intrusions)?’) and one dissociation (‘During run X, did you experience detachment sensations (dissociation)?’) question was assessed per run (Fig. 1).

### Materials

#### Clinician-administered diagnostic interviews

Potential participants were diagnosed with the gold standards of interview-based measures, i.e. the German version of the Structured Clinical Interview for DSM-IV^28^ for Axis I disorders, and the Structured Clinical Interview for Dissociative Disorders.^29^ PTSD diagnosis and symptom levels were interview-assessed by a trained clinician with the Clinician-Administrated PTSD Scale (CAPS-IV^30^).

#### Self-report measures

The participants completed various self-report questionnaires to assess traumatic experiences in childhood (Childhood Trauma Questionnaire (CTQ);^31^ Cronbach's α = 0.95) and lifetime trauma (Essen Trauma Inventory (ETI)^32^), as well as the PTSD Checklist for DSM-IV^33^ (Cronbach's α = 0.72), trait dissociation (German Version of the Dissociative Experiences Scale (FDS);^34^ Cronbach's α = 0.90), depersonalisation (Cambridge Depersonalization Scale (CDS-30);^35^ Cronbach's α = 0.95), somatoform dissociation (Somatoform Dissociation Questionnaire;^36^ Cronbach's α = 0.53), peritraumatic dissociation (Peritraumatic Dissociative Experiences Questionnaire;^37^ authorised German translation by A. Maercker^38^); Cronbach's α = 0.45), trait anxiety (State Trait Anxiety Inventory – Trait Version;^39^ Cronbach's α = 0.62) and depression (Beck Depression Inventory-II;^40^ Cronbach's α = 0.93). On the day of the scan, participants indicated their level of state dissociation (Clinician-Administered Dissociative States Scale (CADSS);^41,42^ Cronbach's α = 0.94) twice, once before entering the MRI scanner and once immediately after exiting the scanner.

#### Responses to Script-Driven Imagery Scale

The Responses to Script-Driven Imagery Scale (RSDI)^27^ is an 11-item self-report scale to assess post-traumatic stress reactions, namely re-experiencing, avoidance and dissociation, in response to SDI during psychobiological symptom provocation research. Participants rated the severity level of each item (e.g. ‘Did you feel disconnected from your body’) on a seven-point Likert from 0 (‘Not at all’) to 6 (‘A great deal’). For the current research, three subscale scores were computed by averaging the sum of self-reported re-experiencing (items 1–4), avoidance (items 5–7) and dissociation symptoms (items 8–11). The latter was employed to test how task-related brain activity corresponds to the acute dissociation experienced during fMRI. In the current sample, the internal consistency of the RSDI scale in the neutral condition was good, with a Cronbach's α of 0.82 for the total scale, 0.45 for the re-experiencing subscale, 0.79 for the avoidance subscale and 0.85 for the dissociation subscale, of which the latter subscale depicted numerically higher internal consistency compared with previous studies by Hopper et al.^17,27^

### Data analysis

#### Script-evoked symptoms

To test whether the experimental manipulation successfully elicited trauma-related symptoms, the patients with PTSD responding to SDI was analysed as follows. First, three paired-sample *t*-tests were performed to compare the responses to SDI, immediately assessed after each condition block inside the scanner, and between the neutral and trauma conditions for each symptom subscale, respectively. Second, a paired-sample *t*-test was employed to compare the mean levels of state dissociation (measured with the CADSS) assessed before and after the neuroimaging paradigm. Third, two separate repeated-measures ANOVAs were employed to test for the impact of repeated presentation (i.e. potential habituation and sensitisation effects) of the autobiographical scripts on dissociation and intrusion, respectively, with script (trauma versus neutral) and repetition (first/second/third run) as within-participant factors.

#### fMRI data acquisition

Participants were scanned on a 3T Siemens Magnetom TrioTim (syngo MR, Siemens, Erlangen, Germany) at the Berlin Institute of Health, Charité Universititätsmedizin Berlin, equipped with a 12-channel hybrid birdcage radiofrequency coil for magnetic resonance signal transmission and reception.

#### Anatomical images

High-resolution T1-weighted anatomic images were acquired using magnetisation-prepared rapid acquisition with a gradient echo sequence scanned in sagittal orientation with the following parameters: field of view (FoV) 256, 192 slices, 1 mm isotropic voxel size, repetition time (TR) 1.9 ms, echo time (TE) 2.52 ms, flip angle 9°, time (TI) 900 ms, 50% distancing factor.

#### Functional images

A set of 40 contiguous, transversely orientated, 3-mm thick functional slices were prescribed parallel to the anterior commissure-posterior plane. The T2*-weighted Blood-oxygen-level-dependent functional brain volumes were obtained using gradient echo planar imaging with an interleaved slice acquisition (64 × 64 matrix size, TR = 2.25 s, TE = 25 ms, flip angle 80°, FoV = 192 mm, distancing factor 20%).

### fMRI data analysis

#### fMRI preprocessing

Image processing and statistical analyses were performed with Statistical Parametric Mapping (SPM for Mac; version 12; Wellcome Department of Neurology, London, UK; https://www.fil.ion.ucl.ac.uk/spm/software/spm12/) within MATLAB 9.9.0 (R2020b; MathWorks for Mac (The MathWorks, Inc., Natick, Massachusetts, United States; see www.mathworks.com/products/matlab.html). DICOM images were converted to NIfTI format with dcm2niix.mac software (version 2 for Mac, 3 November 2020; Chris Rorden, University of South Carolina, South Carolina, United States; see https://github.com/rordenlab/dcm2niix/releases). Field map-derived voxel displacement maps were calculated per session to correct for motion distortions. The functional images corresponding to each condition (neutral versus traumatic imagery) were realigned and unwarped to the mean image. Subsequent preprocessing steps included co-registration of anatomical image to the unwarped mean image, segmentation of the coregistered anatomical image, normalisation (3 × 3 × 3 mm) of the realigned functional images to a Montréal Neurological Institute (MNI) anatomical template and spatial smoothing to a Gaussian kernel of 6 mm full-width half-maximum.

#### First-level analyses

Voxel-wise general linear models were used to investigate the activation patterns during each condition. The following conditions were modelled as regressors: ‘Retrieval’ (27 s) and ‘Re-experiencing’ (33.75 s), present for the sessions ‘Neutral’ and ‘Trauma’. Conditions within the tasks were contrasted voxel-wise to each other in first-level analyses, to identify areas that are less or more active in the trauma (versus neutral) condition at the participant level. The present study used the null periods before (30 s), in-between runs (i.e. 2 min of rest period in between script presentation), and after (30 s) the SDI paradigm as the implicit baseline measures. During these periods, participants had their eyes open staring at a fixation cross instructed to relax and ‘let go’ of the memory material.

#### Second-level analyses

##### Whole-brain analysis

Whole-brain analyses were employed to identify task-elicited activation clusters of contrast trauma versus neutral in a three-step approach following Thome et al.^13^ The main analysis consisted of the whole recall across the script and imagery period, as analysing all volumes acquired during the 1--min period in which participants actively recalled the memory granted maximum statistical power. Then, separate analyses for the retrieval period (during script presentation) and the re-experiencing phase (during imagery) were conducted to investigate potential brain activation differences, as suggested by Thome et al.^13^

##### Region of interest approach

To test the central tenets of conceptualisations of dissociation as the result of limbic overmodulation,^16^ we supplemented our whole-brain analyses with a targeted analysis of amygdala activation using a region-of-interest (ROI) approach. We defined and extracted the bilateral amygdala complex as described in the Juelich Atlas,^43^ employing the JuBrain SPM Anatomy Toolbox for Mac, version 3.0 (Institute of Neuroscience and Medicine (INM-1, INM-7) of the Forschungszentrum Jülich, Germany; see www.fil.ion.ucl.ac.uk/spm/ext/#Anatomy).^44^ Small-volume and Bonferroni corrections were applied to reduce the risk of an inflated type 1 error (Bonferroni-adjusted *P* < 0.025, two-tailed).

##### Association with dissociative processing

To test whether the observed activation differences were (in part) being driven by the increased dissociation during the trauma condition, SPM voxel-wise multiple regression analyses were repeated with covaried dissociation measures. As such, participants’ ratings on acute (RSDI dissociation subscale) and trait dissociation (FDS) and depersonalisation scores (CDS) were separately entered as covariates in the second-level analyses, to identify neural correlates of dissociative processing (whole brain, i.e. inside and outside the previously identified clusters). For the ROI analyses, mean activation parameters of the identified ROIs were extracted per participant, and correlated with their respective dissociation scores (i.e. bivariate correlation, two-tailed).

##### Statistical thresholding

Probabilistic threshold-free cluster enhancement (pTFCE; ^45^) with family-wise error (FWE) correction was employed for statistical thresholding and multiple comparisons correction (*P <* 0.05) for the identification of activation differences by condition. However, given the nature of the phenomenon studied in this investigation, we did not expect very strong brain activation correlates for post-traumatic dissociation. In line with the existing literature of dissociation, in a second step we thresholded the second-level analyses with dissociation scores as covariates at *P <* 0.001 (uncorrected, cluster extent *k* ≥ 10). This was deemed useful to explore potential effects of small or medium effect size that did not survive the initial conservative thresholding.

##### Exploratory analyses

In case the observed activation patterns were not sufficiently explained by dissociative processing, alternative explanations such as the intensity of avoidance or re-experiencing during data acquisition were explored (see Hopper et al^17^).

## Results

### Descriptive and correlational analyses

Demographic variables, including childhood and lifetime traumatic events, are summarised in Table 1. As expected, because of the targeted inclusion procedure, the sample reported a high frequency of interpersonal childhood trauma (CTQ), which was also predominantly labelled as the participant's worst traumatic experience. On average, participants reported six to seven traumatic events across the lifespan (ETI: mean 6.49, s.d. 2.45, range: 2–14) and exhibited high levels of PTSD severity (CAPS-IV: mean 67.45, s.d. 14.04, range: 40–95), as well as dissociation, depression and anxiety (see Table 2). Most dissociation measures were highly intercorrelated: script-elicited acute post-traumatic dissociative reactions were strongly related with state dissociation assessed after the experiment, depersonalisation and peritraumatic dissociation, but not trait dissociation. Severity of childhood trauma experiences correlated positively with trait dissociation, somatoform dissociation and acute re-experiencing, but not acute dissociation. Additional inspection of the CTQ subscales did not depict significant differential associations between childhood maltreatment type and dissociation levels evoked by the behavioural paradigm. Association strength of large effects was found between self-reported PTSD severity and trait, somatoform and peritraumatic dissociation.

**Table 2:** Descriptive and correlational statistics

Variable	*n*	Mean	s.d.	1	2	3	4	5	6	7	8	9	10	11	12	13	14	15	16
1. Script-evoked dissociation (RSDI)^a^	51	2.40	1.47	–															
2. Script-evoked re-experiencing (RSDI)	51	4.79	0.92	−0.097	–														
3. Script-evoked avoidance (RSDI)^a^	51	2.45	1.39	0.327*	−0.171	–													
4. State dissociation (CADSS)	51	54.75	52.95	**0**.459**	−0.132	0.161	–												
5. Trait dissociation (FDS)^a^	51	26.74	15.98	0.273	−0.057	0.117	**0**.579**	–											
6. Depersonalisation (CDS)^a^	47	70.77	48.62	**0**.541**	−0.144	0.138	0.805**	0.735**	–										
7. Somatoform dissociation (SDQ)	47	34.34	11.71	0.226	−0.150	0.035	0.668**	0.662**	0.774**	–									
8. Peritraumatic dissociation (PDEQ)^a^	47	22.60	9.59	**0**.518**	−0.103	0.251	**0**.379**	0.420**	0.557**	0.523**	–								
9. Childhood Trauma (CTQ)^a^	48	74.44	21.96	0.166	−0.238	0.325*	0.195	0.316*	0.235	**0**.412**	0.223	–							
10. Self-reported PTSD severity (PCL)^a^	51	37.92	6.58	0.270	0.023	0.206	0.282*	**0**.586**	**0**.520**	**0**.481**	**0**.531**	0.338*	–						
11. Interview-assessed PTSD severity (CAPS-IV)^a^	51	67.45	14.04	0.335*	−0.017	−0.069	0.150	0.228	**0**.418**	0.284	0.368*	−0.071	0.231	–					
12. Re-experiencing (CAPS-IV Criterion B)^a^	51	19.73	5.47	0.387**	−0.020	−0.004	0.274	0.228	0.339*	0.192	0.187	−0.074	0.098	**0**.712**	–				
13. Avoidance (CAPS-IV Criterion C)^a^	51	25.82	8.19	0.292*	−0.151	0.088	0.302*	0.329*	**0**.497**	0.347*	**0**.460**	0.019	0.251	**0**.691**	0.419**	–			
14. Hyperarousal (CAPS-IV Criterion D)^a^	51	22.82	4.91	0.118	0.267	−0.173	−0.064	0.087	0.130	0.086	−0.039	−0.035	0.164	**0**.581**	**0**.373**	0.197	–		
15. Depression (BDI-II)^a^	48	21.63	13.17	−0.087	0.042	−0.058	0.238	**0**.508**	0.315*	0.361*	−0.128	0.126	0.200	0.152	0.139	0.055	0.266	–	
16. Trait anxiety (STAI-T)^a^	47	55.32	10.50	0.068	−0.013	−0.082	0.324*	**0**.604**	0.523**	**0**.443**	0.178	0.158	**0**.427**	**0**.398**	0.244	0.352*	0.429**	0.623**	–

RSDI, Responses to Script-Driven Imagery Scale; FDS, German Version of the Dissociative Experiences Scale; CDS, Cambridge Depersonalization Scale; SDQ, Somatoform Dissociation Questionnaire; PDEQ, Peritraumatic Dissociative Experiences Questionnaire; CTQ, Childhood Trauma Questionnaire; PCL, PTSD Checklist for DSM-IV; CAPS-IV, Clinician-Administered PTSD Scale – Version IV; BDI-II, Beck Depression Inventory-II; STAI-T, State Trait Anxiety Inventory – Trait Version. *P < 0.05 (two-tailed), **P < 0.01 (two-tailed) in bold.

a. Scores are normally distributed.

### Manipulation check: responses to SDI

Significantly stronger script-evoked dissociation was reported for the trauma (RSDI dissociation subscale mean 13.39, s.d. 5.91) compared with the neutral condition (mean 7.16, s.d. 4.89) (*t*(48) = −7.58, *P* < 0.001), illustrating successful symptom induction during the trauma condition. The successfulness of the paradigm was further supported by significantly increased state dissociation (*t*(49) = −5.08, *P* < 0.001), assessed before (CADSS mean 29.54, s.d. 35.08) and after (mean 54.75, s.d. 52.95) the neuroimaging experiment. The current sample further reported significantly increased re-experiencing (*t*(50) = −21.42, *P* < 0.001) and avoidance (*t*(89) = −7.58, *P* < 0.001) symptoms during the traumatic compared with the neutral condition.

### Repetition effect: dissociation

A significant interaction effect for script by repetition was found (*F*(1.42, 58.42) = 5.31, *P* = 0.013; because of sphericity (Mauchly's test *χ*^2^(2) = 19.50, *P* < 0.001), Greenhouse-Geisser correction was applied). *Post hoc* analyses showed that in the neutral condition, dissociation was not affected by repetition (*F*(1.49, 68.57) = 0.02, *P* = 0.947), whereas repeated presentation of the trauma script significantly increased dissociation levels (*F*(1.67, 76.76) = 5.86, *P* = 0.007). Thus, participants reported consistently low dissociation across repetitions in the neutral condition, but showed a linear increase in dissociation per repetition trial.

### Repetition effect: intrusion

Results indicated no evidence for a similar interaction effect for intrusions (*F*(1.64, 75.64) = 0.63, *P* = 0.504), but significant main effects for script (*F*(1, 46) = 196.59, *P* < 0.001) and repetition (*F*(2, 92) = 3.49, *P* = 0.034). *Post hoc* analyses confirmed that self-reported intrusion was consistently higher in the trauma compared with the neutral condition. No significant *post hoc* tests emerged for repetition in either condition.

### Functional neuroimaging results

#### Dissociation during trauma versus neutral recall

The contrast trauma versus neutral recall (i.e. retrieval and re-experiencing period combined) yielded a large activation cluster centred around the left cerebellum (−6, −70, −25; *t* = 7.24, FWE *P*_pTFCE_ < 0.001, *k* = 4185) including the left inferior occipital cortex (−45, −76, −7), and at the left supramarginal gyrus (−60, −43, 32; *t* = 5.97, FWE *P*_pTFCE_ < 0.001, *k* = 74) (Table 3; see Supplementary Fig. 1 available at https://doi.org/10.1192/bjo.2022.65). Acute post-traumatic dissociation (RSDI dissociation subscale) entered as covariate in the second-level analysis did not depict any significant correlation with brain activation, neither within nor outside the previously identified brain regions. Similarly, covaried analyses with trait dissociation (FDS) or depersonalisation (CDS) did not yield any significant correlations with brain activity.

**Table 3:** Script-elicited brain activity in 51 female patients with post-traumatic stress disorder

*x*	*y*	*z*	*t*	*P*(FWE)	*k*	AAL	Hemisphere
Brain activation clusters during traumatic > neutral recall							
−6	−70	−25	7.24	*P* < 0.001	4185	Lobule VI of cerebellar hemisphere	Left
−45	−76	−7	7.18	*P* < 0.001		Inferior occipital gyrus	Left
0	−61	−28	7.08	*P* < 0.001		Lobule VIII of vermis	
−60	−43	32	5.97	*P* = 0.006	74	Supramarginal gyrus	Left
−57	−34	23	5.69	*P* = 0.015		Supramarginal gyrus	Left
−57	−31	32	5.48	*P* = 0.028		Supramarginal gyrus	Left
Brain activation clusters during traumatic > neutral retrieval							
−45	−82	8	8.21	*P* < 0.001	8863	Middle Occipital gyrus	Left
−54	−64	11	8.19	*P* < 0.001		Middle Temporal gyrus	Left
−60	−58	5	8.09	*P* < 0.001		Middle Temporal gyrus	Left
60	−40	26	6.75	*P* < 0.001	1031	Supramarginal gyrus	Right
−3	−28	−7	6.74	*P* < 0.001		Dorsal raphe nucleus	
63	−28	35	6.68	*P* = 0.001		Supramarginal gyrus	Right
9	5	50	5.87	*P* =0.009	61	Supplementary motor area	Right
−6	8	41	5.70	*P* = 0.014		Middle cingulate and paracingulate gyri	Left
9	11	38	5.54	*P* = 0.023		Middle cingulate and paracingulate gyri	Right
3	−1	62	5.86	*P* = 0.009	52	Supplementary motor area	Right
−6	−7	68	5.66	*P* = 0.016		Supplementary motor area	Left
9	−7	65	5.50	*P* = 0.026		Supplementary motor area	Right
21	−1	−1	5.78	*P* = 0.011	140	Pallidum	Right
42	5	11	5.71	*P* = 0.014		Rolandic operculum	Right
45	14	11	5.67	*P* = 0.016		Inferior frontal gyrus, opercular part	Right
36	11	26	5.76	*P* = 0.012	39	Inferior frontal gyrus, opercular part	Right
−33	11	11	5.39	*P* = 0.036	13	Insula	Left
−39	2	8	5.35	*P* = 0.041		Insula	Left
Brain activation clusters during traumatic > neutral re-experiencing							
–	–	–	–	–	–	–	–


Coordinates (in Montréal Neurological Institute space) and anatomical labels (AAL) for overactivations in patients with post-traumatic stress disorder (PTSD) during the traumatic (versus neutral) memory (a) recall, and (b) retrieval during script presentation; and (c) re-experiencing during imagery (no statistically relevant findings) for a family-wise error (FWE)-corrected threshold of *P*_Probabilistic threshold-free cluster enhancement_ < 0.05, with cluster extent threshold of *k* ≥ 10.

#### Dissociation during trauma versus neutral retrieval

During script presentation (i.e. retrieval of autobiographical memory), participants exhibited significantly increased activation in clusters centred around the left middle occipital and temporal cortex, supramarginal cortex, bilateral supplementary motor area, pallidum and insula (Table 3). Covaried multiple regression models with dissociation measures (RSDI, FDS, CDS) with brain activity (inside and outside of these regions) did not result in any significant findings.

#### Dissociation during trauma versus neutral re-experiencing

No activity clusters could be obtained under the FWE-corrected threshold for the re-experiencing period following script presentation. Likewise, multiple regression analyses with dissociation measures (RSDI, FDS, CDS) as covariates did not result in any significant association with brain activation.

#### ROI analyses

**Fig. 2.**
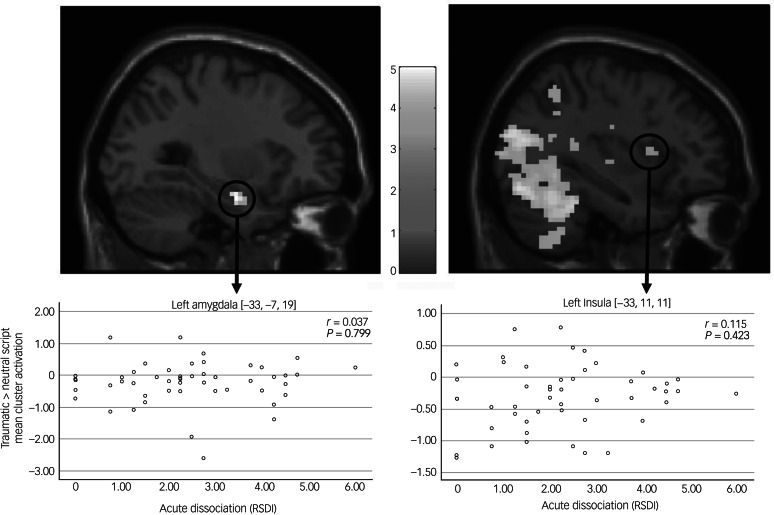
Script-elicited signal activation clusters and corresponding brain-behaviour correlates (*N* = 51).

ROI analyses with small-volume correction for the left and right amygdala complex, as defined by the Juelich Atlas,^43^ yielded increased activity in the left amygdala (−33, −7, 19, *t* = 4.47, *k* = 27, *P*_FWE_ = 0.002) across the whole recall period (see Supplementary Fig. 1). During retrieval, elevated activity was observed in the left amygdala (−33, −7, 19, *t* = 4.98, *k* = 21, *P*_FWE_ < 0.001) and right amygdala (24, −4, −16, *t* = 3.87, *k* = 15, *P*_FWE_ = 0.010). No meaningful activation difference of the bilateral amygdala could be detected during the re-experiencing period alone. Correlational analyses between the derived activation clusters and acute post-traumatic dissociation (see Fig. 2), trait dissociation or depersonalisation symptoms did not yield significant findings. *Post hoc* exploration of a potential association between acute re-experiencing (RSDI) experienced during trauma script presentation and bilateral amygdala activation did not yield significant results.

#### Explorative correlational analyses and stability testing

To test whether the derived activation clusters could be explained by script-evoked acute re-experience or avoidance, the mean scores of these RSDI subscales were separately entered into the second-level models as covariates for the contrast recall, retrieval and re-experiencing period. No activation correlations survived the statistical threshold corrected for multiple testing or emerged at an uncorrected alpha level of *P*_pTFCE_ < 0.001 level with a cluster extent of *k* ≥ 10. To rule out that our contrasts were affected by carry-over effects (i.e. sustained brain activation during the subsequent rest period, see Lamke et al^46^), we employed an additional analysis technique (i.e. ‘scrubbing’^47^) to test the stability of our null results. Results stemming from the adjusted model did not depict meaningful divergences from the previous findings (see Supplementary material).

## Discussion

The current aim was to identify neural correlates of acute post-traumatic dissociation. To this end, brain activation was assessed during SDI in a large sample of female patients with PTSD with a history of interpersonal childhood trauma, and the derived activation was correlated with dissociation severity. The paradigm successfully induced dissociative and intrusive processing during the trauma condition, with increasing intensity per repetition as reported by the participants themselves (see Fig. 1). A subsequent state dissociation assessment confirmed a significant increase of dissociation severity following the paradigm. On the brain level, activation in the cerebellum, inferior occipital gyrus, left supramarginal gyrus and left amygdala significantly increased during traumatic memory recall compared with neutral memory recall. These regions are included in a wider network of brain regions subserving autobiographic memory recall.^48^ Taken together, these results suggest that the SDI paradigm was implemented successfully. However, inspection of neural correlates via covaried multiple regression analyses of the task-elicited brain activity with symptom measures of acute and trait dissociation/depersonalisation did not yield any meaningful results. We further inspected potential activation differences underlying retrieval (i.e. memory script presentation) and re-experiencing (i.e. imagery following script presentation). During trauma retrieval, the contrast yielded widespread elevated brain activation including the middle occipital gyrus, supramarginal gyrus, supplementary motor area, insula and amygdala, whereas no meaningful activation clusters survived statistical thresholding in the trauma re-experiencing period. Again, no distinct associations with dissociation severity emerged.

Post-traumatic dissociation is commonly conceptualised as a reactive defensive mechanism characterised by strong top-down inhibitory control to dampen a hypervigilant innate alarm system and numb physical and emotional distress often elicited by traumatic reminders, threat cues or stressors.^49,50^ On a neurobiological level, excessive medial prefrontal activity is assumed to inhibit limbic regions (e.g. amygdala and insula) and alter processing in circuits involved in automatic physical responding and psychophysiological adaptations located in the brain stem. Present findings based on the brain activity of patients with PTSD reacting to personalised trauma memory scripts were unable to support the notion that post-traumatic dissociation corresponds with the proposed neural underpinnings of (pathological) emotional underengagement, namely excessive medial frontal hyperactivation and concurrent cortico-limbic hypoactivation (e.g. Lanius et al^16^ and Hopper et al^17^). Although a ROI analysis suggested increased activity during trauma confrontation of the amygdala, a region associated with the processing of threat cues and trauma-specific stimuli,^51,52^ the bilateral activation clusters were not large in volume or activation strength and did not correlate with any of the dissociation measures. Notably, none of the current analyses could replicate previous SDI studies indicating increased neural activity in the medial and frontal gyri^18^ and medial prefrontal cortex,^17^ all correlating significantly with acute dissociation.

The lack of identified neural correlates highlights the difficulty to extract reliable biomarkers for subtle subjective experiences such as dissociation via fMRI, especially when encountered in a heterogeneous psychiatric condition as PTSD. Considering that the present study tested almost the same number of patients with PTSD as the three aforementioned SDI studies together,^17–19^ one could argue that previous significant findings are mostly spurious because of small sample sizes analysed with low statistical thresholds, which increase the risk for type 1 errors.^25^ Alternatively, differences in data analyses approaches (e.g. ROI versus whole-brain analyses) or sample characteristics may partially account for the differential findings. For instance, Hopper et al's^17^ sample suffered predominantly from a motor vehicle accident (as opposed to the current sample experiencing interpersonal childhood trauma) and reported numerically lower levels of trait dissociation (Dissociative Experiences Scale mean 9.3), but comparable levels of script-elicited acute dissociation (RSDI dissociation subscale mean 2.12). Childhood trauma, especially sexual and physical abuse, depicted a robust association with dissociation in both general population and psychiatric samples,^53,54^ and has been related to alterations in neurobiological development.^55^ Taking into account that the majority of the current sample reported moderate-to-severe levels of interpersonal traumatic childhood experiences, it is conceivable that the prolonged early stress may have indirectly affected (neural) responses to SDI. However, it should be noted that the severity of childhood trauma correlated significantly with trait and somatoform dissociation, but maltreatment sum (and subscale) scores were unrelated to the acute dissociation experienced during the paradigm in the current sample (see Table 2).

With regard to the SDI paradigm, the current analyses revealed altered neural activity patterns that have already been observed by previous research. The existing PTSD literature has proposed various neural markers of intrusive re-experiencing, hyperarousal and hypervigilance, most prominently overactivation in brain regions related to emotional (e.g. amydgala), visual (e.g. occipital and parietal cortex) and memory processing (e.g. hippocampus) concurrent with a deficient hypoactivation in medial-frontal emotion regulation areas.^16,56,57^ Again, the present findings did not find evidence for altered brain activity in frontal emotion regulation centres. Instead, the current sample depicted widespread elevated activity in the occipital cortex, posterior cerebellum and supramarginal gyrus when contrasting traumatic and neutral memory recall in patients with PTSD. Activation of the occipital cortex has traditionally been associated with both vivid imagery as well as autobiographic memory recall (see meta-analytic reviews by Winlove et al^58^ and Svoboda et al^48^). New studies also implemented such activations in motor imagery.^59^ It is conceivable that elevated brain activity in these regions following traumatic (versus neutral) memory recall corresponds to stronger sensory imagination, including recall of motor actions. Meanwhile, previous meta-analyses of symptom provocation studies and traumatic autobiographical memory recall in PTSD did not report such strong activation differences in the primary visual cortex.^13,60^ Because of the inconsistent findings, it is currently unclear whether enhanced visual processing in the occipital and parietal regions, possibly associated with increased enhanced vividness and sensory imagination, are indeed neural markers of PTSD aetiology as postulated by Patel et al.^57^

The current study further sought to explore the activation differences underlying the recall of autobiographical memories: recall (retrieval and re-experiencing), retrieval only and re-experiencing only. According to a meta-analytical review by Thome et al,^13^ within-group trauma-related recall in patients with PTSD elicited robust activation clusters in proximity to the precentral gyrus, caudate nucleus and right ventromedial prefrontal gyrus across the whole recall period. Likewise, enhanced activation in the ventromedial prefrontal cortices, supramarginal gyrus, precentral gyrus and caudate nucleus was found during retrieval; the latter two are suggestive an ‘increase in procedural-based memory storage of patient's trauma memory’.^13^ In contrast, the main activation clusters during re-experiencing were centred around the right insula (including amygdala and parahippocampal gyrus) and caudate nucleus, which correlated significantly with PTSD severity and may be indicative of enhanced (implicit) memory processing for (non-)associative fear responses during trauma imagery. However, these previous findings suggesting differential brain activation dependant on whether participants listen to the script or asked to imagine the traumatic event are based on a conjunction of studies employing different designs and contrasts. To our knowledge, the present investigation is one of the first to report findings across different processing stages within a single sample, and could only find limited support for the notion that distinct phases depicted differential brain activation.

Unexpectedly, the trauma re-experiencing period, which was the main contrast of interest in previous neuroimaging SDI studies,^13^ elicited fewer and weaker brain activation clusters compared with the trauma retrieval phase. During script presentation (i.e. retrieval), the current sample depicted the strongest and most widespread activity in various brain regions (including the occipital gyri, supramarginal gyrus, supplementary motor area and amygdala), whereas no activation clusters survived statistical thresholding during re-experiencing, and were therefore not reported. It is conceivable that the present sample did not comply sufficiently with the instructions and stopped reliving their traumatic memories once the script stopped playing. This notion remains purely speculative as self-report measures during the paradigm after each block only assessed mean symptom levels across the whole recall period, and not each retrieval and re-experiencing period separately. Still, the overall self-reported data indicated that the manipulation via SDI was successful, as participants reported higher PTSD symptom levels in the trauma compared with the neutral condition, and dissociation increased significantly per repetition of the traumatic script (i.e. sensitisation effect). In conjunction, current and previous findings do indicate that SDI is a valid paradigm to elicit trauma-related symptoms on a subjective level, but it still remains uncertain whether these symptom patterns correspond with robust and symptom-specific brain activation patterns, especially with regard to post-traumatic dissociation.

### Limitations

The current study presented data on the largest sample of patients with PTSD studied with the standard symptom provocation paradigm to date. However, the paradigm itself comes with certain limitations. Most importantly, it is correlational in nature, meaning that although it ensures that the symptom of interest is induced during data acquisition, making a causal attribution more plausible, it still relies on a correlational association between the subjective severity of this symptom and brain activation. An experimental manipulation allowing causal attribution might be able to move beyond this limitation, but is difficult to implement ethically. Another limitation of the present study is the lack of a trauma-exposed control group, which might have allowed us to determine whether the observed activation patterns are specific to PTSD development or are generally associated with exposure to a traumatic event. In addition, the current investigation did not assess whether participants had undergone trauma-focused treatment targeting the intrusive memory used in the script. One could argue that individual differences in habituation to trauma reminders (e.g. during exposure) may explain the intra-participant variability encountered across participants in their reactivity toward trauma script. However, in this case, the level of trauma-elicited reactivity should have correlated with self-reported PTSD symptoms, which was not the case in the present study. Notably, the homogeneity of the current patient sample can be regarded as both strength and weakness, as the focus on female participants with childhood trauma omits the need to control for gender and trauma type in the analyses, but also hampers the generalisation of our findings to other PTSD samples.

To conclude, the present study was unable to identify neural correlates of acute dissociation with the use of SDI and functional neuroimaging. Future research should explore alternative methods with a higher temporal resolution, such as electroencephalography, to analyse intra-participant variability over time (e.g. by using a sliding window approach to study shifts in of association networks over the course of the SDI paradigm) and test alterations of the study design (e.g. counterbalancing of order and comparison of short time intervals before, during and after trauma exposure, to explore potential anticipatory and carry-over effects). It would require an imaging method of both high spatial and temporal resolution to capture how brain patterns in people with PTSD differ within the milliseconds following trauma exposure; for instance, if people experiencing more dissociation depict increased bottom-up activation of prefrontal areas immediately followed by a top-down shutdown response of the initially elevated amygdala-steered threat response.^50^ Additionally, we encourage inspection of brain activation patterns in stratified samples differing in trauma load (i.e. childhood maltreatment types and severity,^61^ multiple lifetime traumatic events, time since trauma onset) and current symptom severity. Arguably, PTSD profile differentiation based on patient history and individual item scores (instead of categorical diagnostic assessments) may lead to more precise brain-behaviour prediction modelling and enhanced ecological validity of theoretical frameworks on post-traumatic dissociation.

## Data Availability

The data that support the findings of this study are available on request from the corresponding author, J.K.D. The data are not publicly available due to restrictions their containing information that could compromise the privacy of research participants.

## Supplementary material

For supplementary material accompanying this paper visit http://doi.org/10.1192/bjo.2022.65. click here to view supplementary material
