# Supplementary material for: Neural correlates of acute post-traumatic dissociation: a functional neuroimaging script-driven imagery study
Source: BJPsych Open. 2022 Jun 10;8(4):e109. doi: 10.1192/bjo.2022.65 (PMC9230559; doi:10.1192/bjo.2022.65)
Supplement: Supplementary file 1 [file S2056472422000655sup001.docx]

**Supplementary Material**

Supplement A

**Suppl. Figure 1**. (a) BOLD responding to traumatic (vs. neutral) recall in left cerebellum [-6, -70, -25; t = 7.24, z = 5.96, p_FWE_ < .001, k = 4185] including the left inferior occipital cortex [-45, -76, -7] and the left supramarginal gyrus. (b) Increased activation of the left amygdala [-33, -7, 19, t = 4.47, k = 27, p_FWE_ = .002] during trauma vs neutral recall (*N* = 51).

(a) (b)


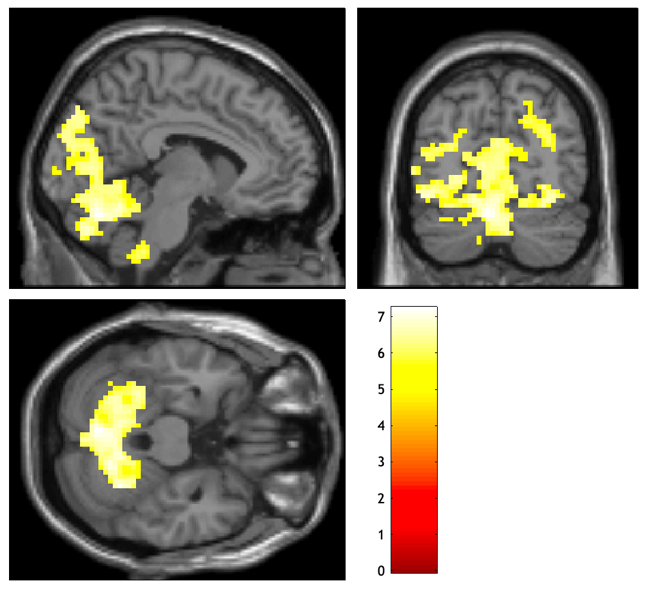

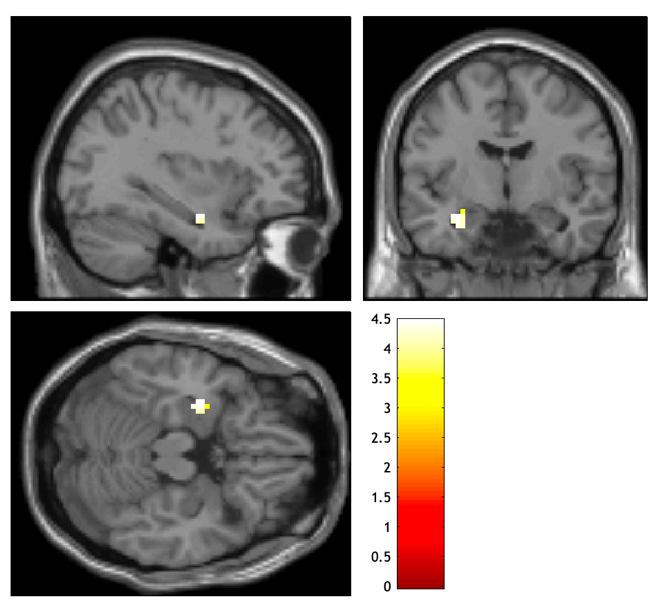


**Suppl. Figure 2**. (a) BOLD activation cluster responding to traumatic (vs. neutral) retrieval centered around left middle occipital gyrus [-45, -82, 8; t = 8.21, z = 6.50, p_FWE_ < .001, k = 8863]. (b) Elevated activation found in the left insula during traumatic (vs. neutral) retrieval [-33, 11, 11; t =5.39, z = 4.77;, p_FWE_ =.013, k = 13].

(a)

**
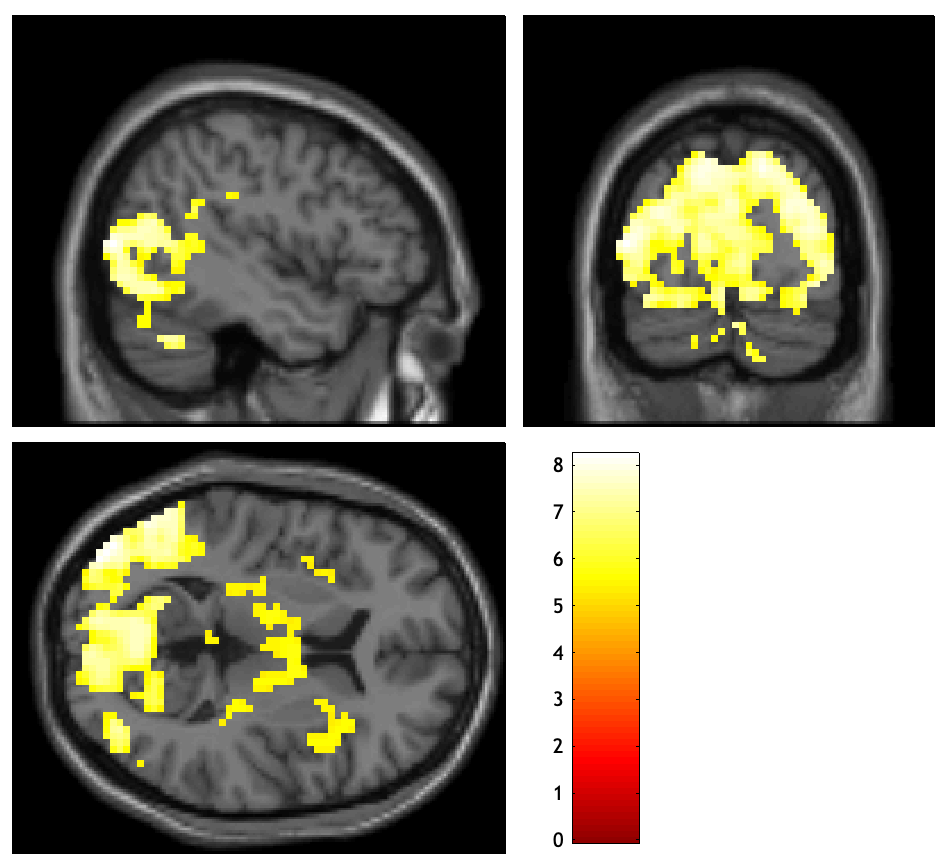
**

(b)

**
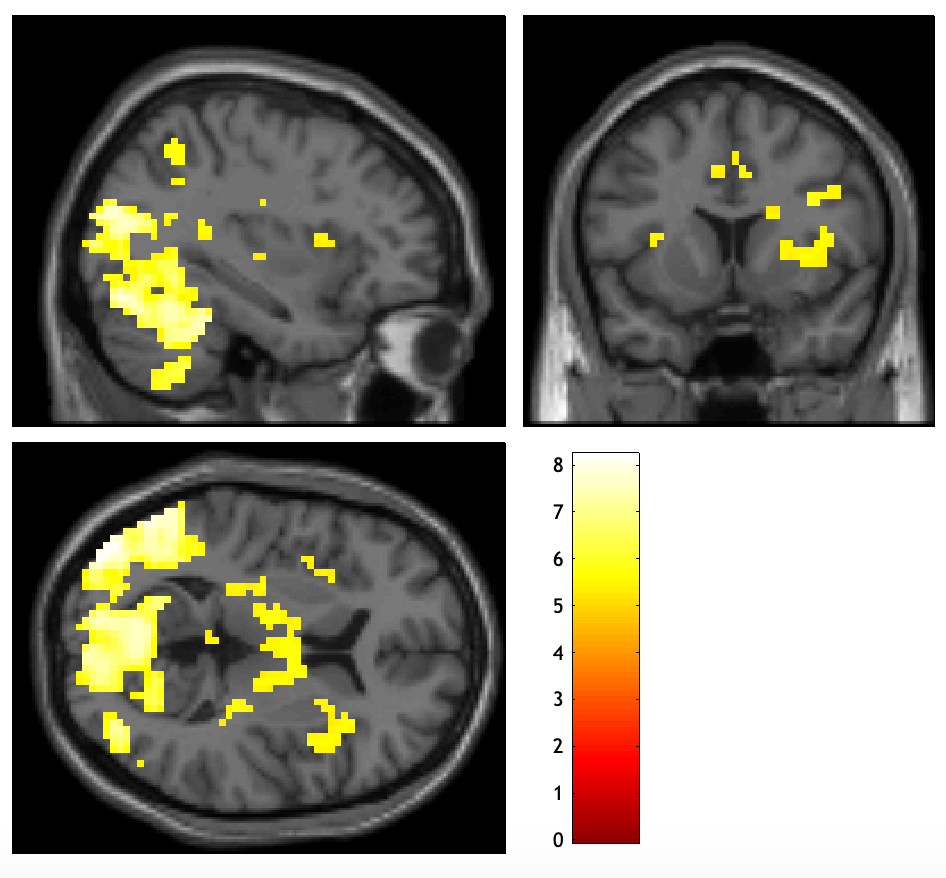
**

Supplement B

This supplement reports brain activation clusters derived from general linear model including scrubbing (see original article cf. **Explorative correlational analyses and stability testing)**

**Stability Testing.**

To rule out that our contrasts were impacted by carry-over effects (i.e., sustained brain activation during the subsequent rest period, see (1)), we employed an additional analysis technique to test the stability of our null results. To this end, we repeated all analyses by adding confound identity matrices as multiple regressors into the general linear model to ignore certain measurement points in the general linear models and thus not assume stability and similarity across trials of brain activity patterns (i.e., ‘scrubbing’; (2)). Scrubbing was employed for the first minute of rest following script presentation and the individual subject delay to initialize the next script presentation to account for potential carry-over effects or anticipation effects, respectively. Results stemming from the adjusted model did not depict meaningful divergences from the previous findings as can be seen in the following Supplementary Tables 1 – 3.

**Suppl. Table 1:** Coordinates (in MNI space) and anatomical labels (AAL^REF^) for over-activations in *N* = 51 female subjects with posttraumatic stress disorder (PTSD) during traumatic (vs. neutral) autobiographical memory recall. Since no activation clusters survived the FWE-corrected threshold of p_pTFCE_ < .05, the table displays activation clusters obtained at uncorrected threshold of p < .001 with cluster extent threshold of k ≥ 10.

| **Traumatic > Neutral Recall (incl. scrubbing)** | | | | | | | |
| --- | --- | --- | --- | --- | --- | --- | --- |
| **x** | **y** | **z** | **T** | **p(unc)** | **k** | **AAL** | **hemisphere** |
| -51 | -73 | -7 | 4.71 | p < .001 | 715 | Inferior occipital gyrus | left |
| -33 | -67 | 17 | 4.58 | p < .001 |  | Middle occipital gyrus | left |
| -33 | -82 | 20 | 4.51 | p < .001 |  | Middle occipital gyrus | left |
| 3 | -64 | -28 | 4.37 | p < .001 | 621 | Lobule VIII of vermis |  |
| -3 | -76 | -34 | 4.26 | p < .001 |  | Crus II of cerebellar  hemisphere | right |
| 6 | -61 | -19 | 4.22 | p < .001 |  | Lobule IV, V of vermis |  |
| 27 | -64 | 32 | 4.32 | p < .001 | 325 | Superior occipital gyrus | right |
| 30 | -73 | 20 | 4.01 | p < .001 |  | Middle occipital gyrus | right |
| 39 | -73 | 20 | 3.92 | p < .001 |  | Middle occipital gyrus | left |
| 33 | -67 | -13 | 4.19 | p < .001 | 105 | Fusiform gyrus | right |
| 36 | -85 | -10 | 4.00 | p < .001 |  | Inferior occipital gyrus | right |
| 30 | -94 | -1 | 3.74 | p < .001 |  | Middle occipital gyrus | right |
| 6 | -28 | -16 | 3.59 | p < .001 | 34 | Red Nucleus | right |
| -3 | -28 | -7 | 3.45 | p < .001 |  | Raphe nucleus, dorsal |  |
| -18 | -49 | 23 | 3.55 | p < .001 | 16 | Cuneus | left |
| -3 | -70 | 17 | 3.43 | p < .001 | 13 | Calcarine fissure and  surrounding cortex | left |

**Suppl. Table 2:** Coordinates (in MNI space) and anatomical labels (AAL^REF^) for over-activations in patients with posttraumatic stress disorder (PTSD) during traumatic (vs. neutral) memory script presentation (i.e. retrieval). FWE-corrected threshold of p_pTFCE_ < .05 at the voxel level with cluster extent threshold of k ≥ 10 with pTFCE.

| **Traumatic > Neutral Retrieval (incl. scrubbing)** | | | | | | | |
| --- | --- | --- | --- | --- | --- | --- | --- |
| **x** | **y** | **z** | **T** | **p(FWE)** | **k** | **AAL** | **hemisphere** |
| -45 | -82 | 8 | 6.20 | 0.003 | 193 | Middle occipital gyrus | left |
| -45 | -79 | -1 | 5.84 | 0.010 |  | Middle occipital gyrus | left |
| -51 | -73 | -7 | 5.77 | 0.012 |  | Inferior occipital gyrus | left |
| 33 | -70 | -13 | 6.08 | 0.005 | 32 | Fusiform gyrus | right |
| 27 | -64 | 32 | 5.88 | 0.009 | 118 | Superior occipital gyrus | right |
| 27 | -85 | 23 | 5.80 | 0.011 |  | Superior occipital gyrus | right |
| 42 | -82 | 14 | 5.60 | 0.021 |  | Middle occipital gyrus | Right |
| 3 | -64 | -25 | 5.86 | 0.009 | 142 | Lobule VI of vermis |  |
| -9 | -70 | -28 | 5.85 | 0.010 |  | Crus I of cerebellar  hemisphere | Left |
| -6 | -58 | -25 | 5.74 | 0.013 |  | Lobule VI of vermis |  |

**Suppl. Table 3:** Coordinates (in MNI space) and anatomical labels (AAL^REF^) for over-activations in patients with posttraumatic stress disorder (PTSD) during traumatic memory re-experiencing compared to neutral re-experiencing. As no activation clusters survived the FWE-corrected threshold of p_pTFCE_ < .05, the table displays activation clusters obtained at uncorrected threshold of p < .001 with cluster extent threshold of k ≥ 10 without pTFCE.

| **Traumatic > Neutral Re-experiencing (incl. scrubbing)** | | | | | | | |
| --- | --- | --- | --- | --- | --- | --- | --- |
| **x** | **y** | **z** | **T** | **p(unc)** | **k** | **AAL** | **hemisphere** |
| -42 | -64 | 14 | 4.08 | p < .001 | 32 | Middle Temporal | left |
| -33 | -67 | 17 | 4.01 | p < .001 |  | Middle Occipital | left |
| -33 | -82 | 20 | 3.81 | p < .001 |  | Middle Occipital | left |

**Dissociation Correlates (including scrubbing)**

No significant correlations were detected between the dissociation measures of script-evoked acute dissociation (RSDI Dissociation subscale), trait dissociation (FDS-20), and depersonalization (CDS) and brain activity detected inside or outside the derived activation clusters during from traumatic (vs. neutral) recall, retrieval or re-experiencing stemming from the adjusted analyses model.

**References**

1. Lamke JP, Daniels JK, Dörfel D, Gaebler M, Rahman RA, Hummel F, et al. The impact of stimulus valence and emotion regulation on sustained brain activation: Task-rest switching in emotion. PLoS One. 2014 Mar 28;9(3).

2. Power JD, Barnes KA, Snyder AZ, Schlaggar BL, Petersen SE. Spurious but systematic correlations in functional connectivity MRI networks arise from subject motion. Neuroimage [Internet]. 2012 Feb 1 [cited 2021 Oct 8];59(3):2142. Available from: /pmc/articles/PMC3254728/
